# Combining [^177^Lu]Lu-DOTA-TOC PRRT with PARP inhibitors to enhance treatment efficacy in small cell lung cancer

**DOI:** 10.1007/s00259-024-06844-1

**Published:** 2024-07-18

**Authors:** Hartmut Rauch, Carolin Kitzberger, Kirti Janghu, Pavithra Hawarihewa, Nghia T. Nguyen, Yu Min, Simone Ballke, Katja Steiger, Wolfgang A. Weber, Susanne Kossatz

**Affiliations:** 1 Department of Nuclear Medicine, TUM School of Medicine and Health, University Hospital Klinikum Rechts Der Isar, and Central Institute for Translational Cancer Research (TranslaTUM), Technical University of Munich, Munich, Germany; 2 Comparative Experimental Pathology (CEP), Institute of Pathology, School of Medicine and Health, Technical University of Munich, Munich, Germany; 3 Department of Chemistry, TUM School of Natural Sciences, Technical University Munich, Munich, Germany

**Keywords:** PARP inhibitors, PRRT, Radioligand therapy, SSTR2, Small cell lung cancer

## Abstract

**Purpose:**

Small cell lung cancer (SCLC) is a highly aggressive tumor with neuroendocrine origin. Although SCLC frequently express somatostatin receptor type 2 (SSTR2), a significant clinical benefit of SSTR2-targeted radionuclide therapies of SCLC was not observed so far. We hypothesize that combination treatment with a PARP inhibitor (PARPi) could lead to radiosensitization and increase the effectiveness of SSTR2-targeted therapy in SCLC.

**Methods:**

SSTR2-ligand uptake of the SCLC cell lines H69 and H446 was evaluated in vitro using flow cytometry, and in vivo using SPECT imaging and cut-and-count biodistribution. Single-agent (Olaparib, Rucaparib, [^177^Lu]Lu-DOTA-TOC) and combination treatment responses were determined in vitro via cell viability, clonogenic survival and γH2AX DNA damage assays. In vivo, we treated athymic nude mice bearing H69 or H446 xenografts with Olaparib, Rucaparib, or [^177^Lu]Lu-DOTA-TOC alone or with combination treatment regimens to assess the impact on tumor growth and survival of the treated mice.

**Results:**

H446 and H69 cells exhibited low SSTR2 expression, i.e. 60 to 90% lower uptake of SSTR2-ligands compared to AR42J cells. In vitro, combination treatment of [^177^Lu]Lu-DOTA-TOC with PARPi resulted in 2.9- to 67-fold increased potency relative to [^177^Lu]Lu-DOTA-TOC alone. We observed decreased clonogenic survival and higher amounts of persistent DNA damage compared to single-agent treatment for both Olaparib and Rucaparib. In vivo, tumor doubling times increased to 1.6-fold (H446) and 2.2-fold (H69) under combination treatment, and 1.0 to 1.1-fold (H446) and 1.1 to 1.7-fold (H69) in monotherapies compared to untreated animals. Concurrently, median survival was higher in the combination treatment groups in both models compared to monotherapy and untreated mice. Fractionating the PRRT dose did not lead to further improvement of therapeutic outcome.

**Conclusion:**

The addition of PARPi can markedly improve the potency of SSTR2-targeted PRRT in SCLC models in SSTR2 low-expressing tumors. Further evaluation in humans seems justified based on the results as novel treatment options for SCLC are urgently needed.

**Graphical Abstract:**

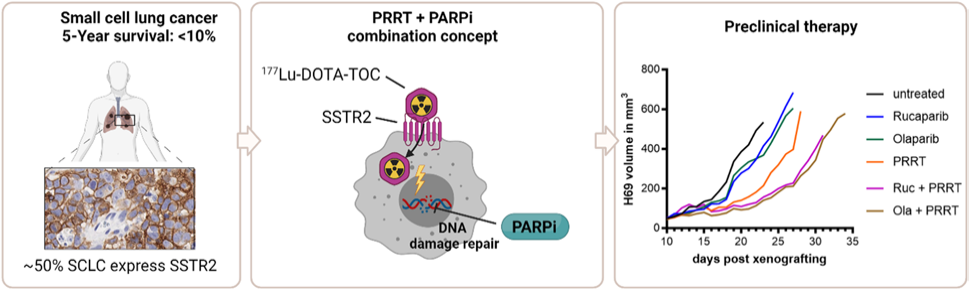

**Supplementary Information:**

The online version contains supplementary material available at 10.1007/s00259-024-06844-1.

## Introduction

Small cell lung cancer (SCLC) is a highly aggressive tumor associated with early metastasis formation and – despite often a good response to initial therapy – a 5-year survival rate of less than 10% [1]. For limited-stage disease, the current standard of care is based on concurrent chemoradiotherapy, mainly by combining platinum-based chemotherapy with external-beam-radiotherapy [2]. In patients with extensive-stage disease, the combination of chemotherapy with immune checkpoint inhibition has modestly improved median overall survival by 2–3 months when compared to chemotherapy alone [2, 3].

An alternative therapeutic approach for extended-stage disease could be peptide-receptor radionuclide therapy (PRRT) with somatostatin receptor (SSTR) ligands [4]. The vast majority of SCLC (> 80%) express neuroendocrine markers, and about 50% of all SCLCs express significant levels of somatostatin-receptor type 2 (SSTR2) [5, 6]. Hence, there is a rationale for the use of SSTR2-targeted therapies in SCLC patients. To date, clinical outcomes of retrospective series, which investigated SSTR2-targeted PRRT in SCLC have been rather inconclusive. In one trial, six patients treated with ^90^Y-DOTATOC showed no response as determined via CT scans and showed disease progression with median progression-free survival of 38 days [7]. In another trial of 11 patients treated with either ^90^Y-DOTATOC/DOTATATE or ^177^Lu-DOTATOC/DOTATATE none showed clinical or radiographic response with disease progression approximately 48 days after PRRT [8]. A recent study selected 14 patients for ^177^Lu-DOTATOC/DOTATATE treatment through somatostatin receptor imaging from a cohort of 67 patients. Of these 14 patients, 4 patients showed stable disease and 1 showed partial remission, while 7 showed progressive disease, representing the so far most encouraging study [9]. Uptake of SSTR2 ligands in SCLC is typical lower than in NETs which correlates with lower expression of SSTR2 on immunohistochemical assessment [9–13]. This finding, combined with the rapid proliferation of SCLCs could explain the limited effectiveness of PRRT in SCLC.

The Poly-ADP-ribose-polymerase 1/2 (PARP) enzymes plays a major role in many cellular DNA damage repair pathways [14, 15]. Over the last few years, different PARP1/2 inhibitors (PARPi) have been approved for treatment of several malignancies, including subgroups of ovarian, breast and prostate cancer [16, 17]. These approvals have largely been limited to cancer with homologous recombination (HR) deficiencies such as conveyed by BRCA1/2 mutations [18]. However, there is also preliminary evidence that PARPi can act as radio- and chemosensitizers in non-HR deficient cells [19, 20]. Currently, several clinical studies are investigating the combination of Olaparib and SSTR2-targeting PRRT in NETs (NCT04086485, NCT05870423, NCT05053854).

In this study, we investigated if treatment with the PARPi Olaparib and Rucaparib can improve the therapeutic efficacy of SSTR2-targeted PRRT in different SCLC models in vitro and in vivo. We hypothesized that by inhibiting cellular DNA damage repair, cells can be sensitized to radiation damage and thus enable more efficacious PRRT in SSTR2 low-expressing SCLC.

## Materials and methods

### Preparation of [^177^Lu]Lu-DOTA-TOC

[^177^Lu]Lu-DOTA-TOC (“PRRT”) was readily prepared by ITM according to clinical production standards with an average molar activity of 223 MBq/nmol. Radiochemical purity (rcp) was assayed via radio-TLC (AR-2000, Bioscan) to ensure > 97% rcp for in vivo use. [^177^Lu]Lu-DOTA-TOC was diluted in RPMI-1640 medium (Gibco, #61870036) for in vitro and in 0.9% NaCl (B. Braun) for in vivo use. Chemicals and solvents were sourced from ITW Reagents, Sigma Aldrich and Avantor.

### Preparation of PARPi

For in vitro assays, the PARPi (Olaparib, Rucaparib; MedChemExpress, #HY-10162, #HY-10617A) were prepared as 20 mM or 100 mM stock solutions in dimethyl sulfoxide (DMSO) and subsequently diluted in medium. All treatment and control (“untreated/medium”) conditions were adjusted to contain the same final DMSO (max. 0.5% (v/v)) concentration to ensure that observed treatment effects were not DMSO-related. For in vivo use, stock solutions of Olaparib and Rucaparib in DMSO were prepared at a concentration of 75 mg/mL, which were diluted with PEG300 (MedChemExpress, #HY-Y0873) and PBS to yield a final concentration of 1.5 mg/mL (2% DMSO, 30% PEG300 in PBS) directly before use.

### Synthesis of a fluorescent sst-analog

A fluorescent sst-antagonist containing SulfoCy5 (Jena Bioscience; CLK-A130-5) was synthesized using strain-promoted azide-alkyne cycloaddition (SPAAC) as described previously [21]. Briefly, azide functionalized peptide (0.5 mg, 0.27 µmol) was dissolved in 1 mL dry DMSO. DBCO-SulfoCy5 (0.5 mg, 1.07 µmol) was added to the reaction mixture, stirred at 37 °C for 3 h, then at room temperature overnight. The product “Fluo-Oct” was purified by HPLC with H_2_O/MeCN + 0.1% TFA as eluent, yielding 0.65 mg (0.23 µmol, 84%; Suppl. Figure 1). For flow cytometry, a 1 mM stock solution in DMSO was prepared.

### Cell culture

The human SCLC cell lines H69, H446 and rat pancreatic cancer cells AR42J (all ATCC) were cultured in RPMI-1640 growth medium with 10% fetal bovine serum (FBS, Gibco, #A5256801) and 1% penicillin and streptomycin solution (Pen-strep, Gibco, #15140–122) using standard aseptic technique. Cells were maintained in an incubator at 37 °C in 5% atmospheric CO_2_ concentration and were passaged every 3–4 days. Cells were regularly subjected to mycoplasma testing, which was always reported negative.

### Flow cytometry

To investigate the binding of the fluorescent octreotide analog (Fluo-Oct) to the SSTR2-positive cell lines AR42J, H69, and H446, cells were prepared in a concentration of 1 × 10^6^ cells/mL in ice-cold FACS buffer (PBS with 5% FBS and 0.1% NaN_3_). Cells were incubated with 0.25 µM Fluo-Oct for 30 min at 4 °C in a dark environment. In the blocking group, cells were incubated with 25 µM octreotide (MedChemExpress, #HY-17365) for 15 min at 4 °C, followed 0.25 µM Fluo-Oct for 15 min in the dark. Then, cells were stained with 0.5 µg/mL DAPI (Biolegend, #422801) for 5 min before flow cytometry analysis on a BD FACSCanto instrument. Data were analyzed using FlowJo software.

### Cell viability assessment

Cell viability was determined using the AlamarBlue high sensitivity (HS) assay (Thermo Fisher Scientific, #A50101). 5,000 cells per well were seeded in 96-well black clear F-bottom plates (H446) or 96-well black clear V-bottom plates (H69). H69 suspension cells were centrifuged and resuspended for each washing step. 24 h after seeding, PARPi (0.1 – 100 µM final concentration per well), [^177^Lu]Lu-DOTA-TOC (10 – 250 kBq final activity per well) or combinations thereof were added to the cells for 24 h ([^177^Lu]Lu-DOTA-TOC) and/or 72 h (PARPi). All wells, including untreated wells, contained 0.5% (v/v) DMSO throughout the assay. 96 h after seeding, medium was removed and resazurin (AlamarBlue HS) solution was added to each well at a final concentration of 10% (v/v) and incubated for 1 h at 37 °C before fluorescence was measured using a Biotek Synergy HT plate reader (Excitation: 540/35 nm, Emission: 590/20 nm). Relative viability was calculated in relation to “untreated” wells. We carried out three biological repeats with 3–6 technical replicates per plate. IC_50_-values were computed by applying the nonlinear regression function (Dose–response – Inhibition; Inhibitor vs. normalized response) in GraphPad Prism 9.

### DNA damage determination (γH2AX)

30,000 H446 cells were seeded into 8-well glass bottom slides (Ibidi, #80807) 24 h before receiving the respective treatments for 1 h (Medium, 1 µM PARPi, 25 kBq [^177^Lu]Lu-DOTA-TOC, 1 µM PARPi + 25 kBq [^177^Lu]Lu-DOTA-TOC). After treatment, cells were either stained immediately for γH2AX foci or subjected to a 23 h post-incubation resting period in medium. For immunofluorescence staining, cells were fixed with 4% PFA and permeabilized with 0.1% TritonX-100/PBS for 10 min before blocking with 3% BSA/PBS for 1 h. Then, cells were incubated with the primary Phospho-Histone H2A.X antibody (1:500; MA5-27753, ThermoFisher) for 1 h, followed by the secondary AlexaFluor-488-conjugated goat-anti-mouse IgG antibody (1:2000; A110001, ThermoFisher) for 1 h. Nuclei were counterstained with 10 µg/mL Hoechst 33,342 (Invitrogen, #H3570) for 5 min. Cells were imaged on an EVOS M7000 microscope (Thermo Fisher Scientific). Quantification of foci per cell was performed using the Software CellProfiler Version 4.2.1 and the “Speckle counting” pipeline.

### Clonogenic assay

H446 cells were seeded in 6-well plates 48 h prior to the experiment. Seeding numbers were adjusted in each treatment group (Medium: 300, 400; PARPi: 400, 600; [^177^Lu]Lu-DOTA-TOC: 600, 1200; combination therapy: 1200, 3000). Cells were treated with medium, 0.5 µM PARPi for 72 h, 12.5 kBq [^177^Lu]Lu-DOTA-TOC for 24 h, or the combination therapy of 0.5 µM PARPi for 72 h and 12.5 kBq [^177^Lu]Lu-DOTA-TOC during the first 24 h. Following treatment, compounds were removed, and fresh medium was added. After 7 days, colonies were fixed with cold methanol, stained with 5% crystal violet in PBS and subsequently counted. Survival fractions were calculated by dividing the plating efficiency as counted colonies divided by seeded cells of the respective treatment group through the plating efficiency of the untreated control group.

### Animal experiments

Authorization for all animal experiments was obtained from the Regierung von Oberbayern. Mice were housed under specific-pathogen free conditions with access to mouse chow and water ad libitum. Female 7–8 weeks old athymic nude mice (Crl:NU/NCr-Foxn1nu, Charles River Laboratories, Sulzfeld) were subcutaneously injected with 1–4 × 10^6^ H69 or H446 cells in 1:1 RPMI-1640 medium:Matrigel (Corning Inc., #11543550). Animals were regularly monitored as defined in the license.

### Biodistribution

After the subcutaneous tumors reached a size of approximately 100–200 mm^3^ animals were injected intravenously with approximately 40 MBq [^177^Lu]Lu-DOTA-TOC with or without prior injection of 1 mg Octreotide for blocking (n = 3/model). After 4 or 72 h, the mice were euthanized and dissected. Organ weights were determined using an analytical balance (Sartorius). A Wizard^2^ gamma counter (PerkinElmer) was used to determine radioactivity in each organ after calibration with dilutions of [^177^Lu]Lu-DOTA-TOC solution.

### SPECT imaging

SPECT imaging was performed for 60 min per animal on a nanoScan SPECT/CT (Mediso) at different timepoints (1 h, 24 h, 72 h) after injection of approx. 25 MBq [^177^Lu]Lu-DOTA-TOC per animal (n = 3/model). Reconstruction, image analysis and quantification of SPECT data and image analysis were performed using Nucline and Interview fusion software (both Mediso).

### *In vivo* treatment study – single PRRT dose

10–15 days after xenografting, mice were randomized into 4 (H446) or 6 (H69) groups after stratification for tumor size to ensure a uniform initial tumor size distribution (H446: ~ 150 mm^3^; H69: ~ 60 mm^3^; Supplementary Fig. 2A, B). Mice in the PARPi and combination therapy groups received daily i.p. injections of PARPi on days 1–5 and 8–12 at a dose of 10 mg/kg. Mice in the [^177^Lu]Lu-DOTA-TOC and the combination therapy groups received a single dose of approx. 40 MBq [^177^Lu]Lu-DOTA-TOC on day 3 of PARPi treatment. Length, width and depth of the subcutaneous tumors were measured daily using a caliper and tumor volume was calculated using the ellipsoid formula. Mice were weighed regularly after start of the treatment. Mice were euthanized when they reached pre-defined criteria. During the study, only the criteria of tumor growth (single dimension ≤ 15 mm) or maximum observation time came into effect. In the H69 cohort, one animal per group was euthanized 3 days after PRRT (or equivalent time) and tumor, bone and kidney tissues were processed for histopathology.

### *In vivo* treatment study – fractionated PRRT dose

In a second treatment study using the H69 model, the single 40 MBq dose of [^177^Lu]Lu-DOTA-TOC PRRT was fractionated into multiple (2 or 3) lower doses of 20 MBq and combined with Olaparib (daily i.p. doses of 10 mg/kg). Prior to the beginning of treatment, mice were randomly assigned to 7 treatment groups (n = 5–6 animals/group) with an initial mean tumor volume of approx. 50 mm^3^ per group (Supplementary Fig. 2C). The following cohorts were investigated: *i)* untreated, *ii)* Olaparib with 2 × dosing cycles on days 1–5 and 7–11, *iii)* Olaparib with 3 × dosing cycles on days 1–5, 7–11 and 13–17, *iv)* 2 × doses [^177^Lu]Lu-DOTA-TOC on days 3 and 9, *v)* 3 × doses of [^177^Lu]Lu-DOTA-TOC on days 3, 9 and 15, *vi)* combination group (2x) receiving Olaparib on days 1–5 and 7–11 and [^177^Lu]Lu-DOTA-TOC on days 3 and 9 of PARPi treatment, and *vii)* combination group (3x) receiving Olaparib on days 1–5, 7–11 and 13–17 and [^177^Lu]Lu-DOTA-TOC on days 3, 9 and 15. Body weight and tumor volumes were determined every 2–3 days. Animals were sacrificed when pre-defined endpoint criteria or humane endpoint was reached. In this study, only the pre-defined endpoint, a tumor volume of 1000 mm^3^ with a maximal size of ≤ 15 mm in a single dimension, was reached. After a maximum observation period of 4 weeks after the last treatment, the study terminated in accordance with the animal protocol.

### Histology

All samples were fixated in 4% PFA for 24 h and subsequently stored in 70% ethanol at 4 °C until the radioactivity had fully decayed. Samples containing bone for bone marrow analysis were decalcified using OSTEOSOFT® (Merck) solution. Formalin-fixed paraffin embedded (FFPE) tissues were cut into 2 µm sections using a microtome. After deparaffinization and rehydration, tissue sections were subjected to standard H&E staining. Tumor sections were additionally subjected to IHC. IHC staining was performed on a Bond RXm (Leica, Nussloch) autostainer using the following primary antibodies: Cleaved Caspase 3 (Clone 5A1E, Cell signaling #9664L; 1:150 dilution), PARP1 (Proteintech #13,372–1-AP, 1:250 dilution), SSTR2 (Clone umb1, abcam #ab134152. 1:150 dilution), γH2AX (pSER139, Novus #NB100-2280, 1:500 dilution) with optimized protocols of the Comparative Experimental Pathology Core Facility. Stained tissue sections were digitalized, and the Aperio ImageScope Software was used for data analysis.

### Statistical analysis

Results are given as mean ± SD, mean ± SEM or percent of control, as stated in the figure caption, respectively. Animal survival of in vivo therapy studies was depicted as Kaplan Meier plots and statistical significance analyzed by log-rank (Mantel-Cox) test. *p*-values < 0.05 were considered as significant. Data visualization and statistical analysis was performed using GraphPad Prism (Version 9.5.1).

## Results

### *In vitro* SSTR2-targeted ligand uptake

**Fig. 1:**
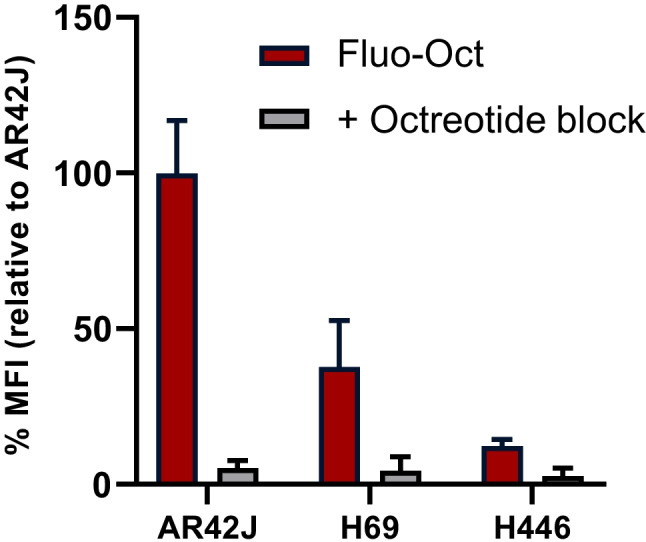
Binding of a fluorescent octreotide analog as determined by flow cytometry in AR42J, H69 and H446 cells with and without prior blocking with octreotide. Values are relative MFI (mean fluorescence intensity) normalized to AR42J. Values represent means and standard deviations of *n* = 3 repeats

We first performed a flow cytometry experiment with a fluorescent octreotide analog to compare the binding of the fluorescently labeled SSTR2-binding peptide in H69, H446 and AR42J cells. Flow cytometric quantification showed that binding to H69 cells was 63% lower than to AR42J cells and 88% lower to H446 cells compared to AR42J cells (Fig. 1). Furthermore, octreotide blocking confirmed SSTR specificity of the binding.

### *In vitro* radiosensitization potential

**Fig. 2:**
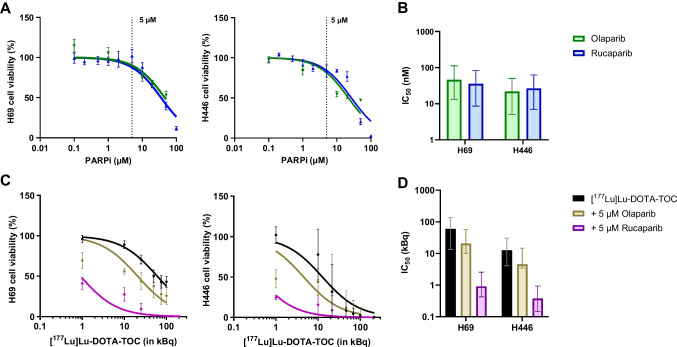
In vitro radiosensitization potential of PARPi in SCLC cells. (A) Cell viability of H69 and H446 cells after treatment with Olaparib or Rucaparib (PARPi) for 72 h. (B) IC_50_-values for treatment of H69 and H446 cells with PARPi. (C) Cell viability of H69 and H446 cells after treatment with [^177^Lu]Lu-DOTA-TOC (24 h) alone or with 5 µM PARPi (72 h). (D) IC_50_-values for [^177^Lu]Lu-DOTA-TOC alone or in combination with PARPi. Values are mean ± SEM (A, C) or mean ± 95% CI from 3 independent experiments (B, D). Fit and IC_50_ were calculated using a nonlinear regression function in GraphPad PRISM

We assessed in vitro if PARPi can sensitize the SCLC cell lines to [^177^Lu]Lu-DOTA-TOC PRRT. First, we determined the response of H69 and H446 to Olaparib and Rucaparib treatment. Both cell lines displayed IC_50_ values in the range of 20–40 µM (Fig. 2A + B). We did not detect differences in PARPi sensitivity between H69 and H446 or between Olaparib and Rucaparib. From these results, we selected 5 µM PARPi as concentration for combination treatments. Combining PARPi with PRRT resulted in IC_50_-values that decreased from 60.6 kBq (PRRT) to 20.7 kBq (+ 5 µM Olaparib) and 0.9 kBq (+ 5 µM Rucaparib) in H69 cells. This is a 2.9-fold and 67-fold reduction, respectively. In H446 cells, IC_50_-values also decreased from 12.7 kBq (PRRT) to 4.5 kBq (+ 5 µM Olaparib) and 0.4 kBq (+ 5 µM Rucaparib), which reflects a 2.8-fold and 32-fold reduction, respectively (Fig. 2C + D).

**Fig. 3:**
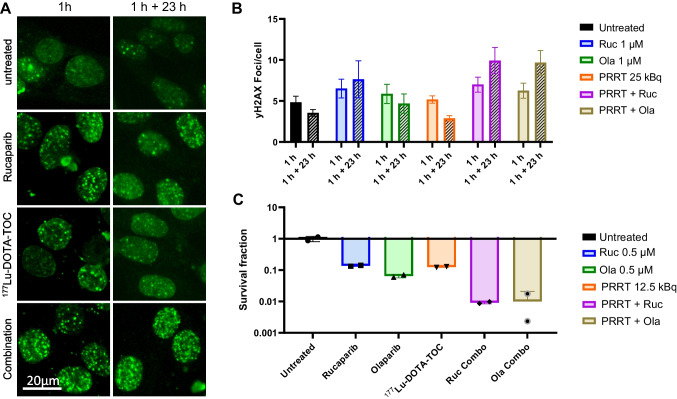
Determination of γH2AX foci formation and clonogenic survival in H446 cells. **(A)** Exemplary images of H446 cells after mono- or combination therapy directly after treatment or after a 23 h post-incubation time. **(B)** Number of γH2AX foci per cell after mono- or combination therapy directly after treatment or after a 23 h post-incubation time in medium. Displayed are mean ± SEM from 3 individual experiments. **(C)** Surviving fractions of H446 cells mono- or combination therapy. Displayed are mean values from 2 independent experiments

Quantification of γH2AX foci in H446 cells showed moderate increases in the mean number of foci per cell after 1 h of treatment compared to untreated cells (Fig. 3A + B). However, after a post treatment resting period of 23 h we observed a further increase in the number of foci in the combination treatment groups, while untreated cells and cells treated with PARPi or [^177^Lu]Lu-DOTA-TOC only showed a persistent or decreasing number of foci 23 h post treatment (Fig. 3B).

We also conducted clonogenic assays using 0.5 µM Olaparib/Rucaparib and 12.5 kBq [^177^Lu]Lu-DOTA-TOC alone or in combination. While the mean surviving fractions ranged between 7% (Olaparib) and 13% (Rucaparib; [^177^Lu]Lu-DOTA-TOC) of untreated cells for the monotherapies, the combination regimens showed only 0.1% surviving fraction in both Rucaparib or Olaparib + [^177^Lu]Lu-DOTA-TOC (Fig. 3C).

### *In vivo* biodistribution studies of SSTR2-targeted ligands in SCLC models

**Fig. 4:**
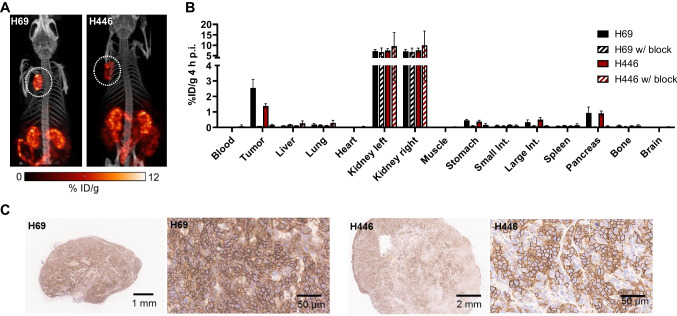
Imaging and biodistribution of [^177^Lu]Lu-DOTA-TOC. **(A)** SPECT/CT images of H69 and H446 tumor-bearing bearing mice 1 h after i.v. injection of ~ 25 MBq [^177^Lu]Lu-DOTA-TOC. White circles indicate tumors. **(B)** Biodistribution of H69 and H446 xenograft bearing mice 4 h after i.v. injection of ~ 40 MBq. [^177^Lu]Lu-DOTA-TOC with and without prior octreotide (1 mg) blocking. Biodistribution values are mean ± SD from n = 3 animals/group. **(C)** Histological sections from H69 and H446 tumors stained for SSTR2 expression (Brown staining)

SPECT imaging showed relatively higher uptake of [^177^Lu]Lu-DOTA-TOC in H69 xenografts compared to H446 (Fig. 4A, Supplementary Fig. 3A, B). We also assessed [^177^Lu]Lu-DOTA-TOC biodistribution (Fig. 4B, Supplementary Fig. 3C). Tumor uptake at 4 h p.i. was rather low at 2.5% ID/g (H69) and 1.5% ID/g (H446), but was highly specific, as seen by octreotide blocking. Hence, in vivo uptake of [^177^Lu]Lu-DOTA-TOC was consistent with the in vitro cell binding experiments (Fig. 1) and confirmed higher uptake in H69 compared to H446 xenografts. We also stained explanted tumors for SSTR2 expression and found higher density and intensity of SSTR2 in H69 compared to H446 tumors (Fig. 4C).

### *In vivo* PRRT in combination with PARPi in SCLC models

**Fig. 5:**
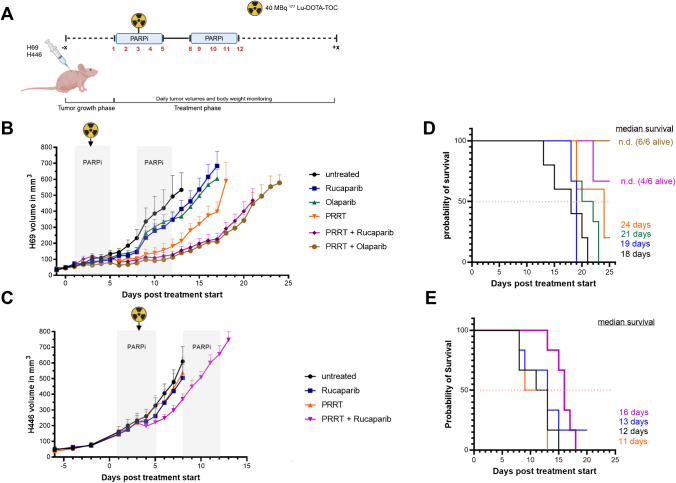
In vivo treatment study for single dose PRRT application. **(A)** Application schedule for PRRT and PARPi treatment in H69 and H446 tumor bearing mice. Tumor volume of H69 **(B)** and H446 **(C)** xenografts. Grey areas depict days of PARPi therapy. Radioactivity sign shows day of PRRT. Tumor volumes are given as mean ± SEM. Curves end when first animal reached endpoint criteria and left group (= reached a tumor size in one dimension of > 15 mm). Kaplan–Meier survival curves of H69 **(D)** and H446 **(E)** tumor bearing mice for single PRRT dose groups. Shown are median survival times. Total observation time after xenografting was 38 days

An overview of the single PRRT in vivo treatment study protocol can be seen in Fig. 5A. In the H69 tumor-bearing mouse model, all therapies showed a trend towards a reduced growth rate compared to the untreated tumors. For both Olaparib and Rucaparib monotherapy, tumor doubling times increased (4.0 days and 4.1 days, respectively) compared to the untreated group (3.6 days), but tumor growth resumed at the beginning of the 2nd week of therapy. For PRRT monotherapy, tumor doubling time increased to 6.4 days, but the most pronounced growth delay occurred in both combination groups, resulting in 8.0 days tumor doubling time for both Olaparib and Rucaparib combination therapy (Fig. 5B, Supplementary Fig. 4A). While a mean tumor volume of 400 mm^3^ was reached 10 days after start of treatment in the untreated group, this was delayed to 13 days (Olaparib, Rucaparib), 17 days (PRRT) and 20 days (both combination therapy groups).

In the H446 model, the tumors treated with either monotherapy continued their growth very similar to the untreated group with doubling times of 3.7 (PRRT) or 3.9 (PARPi) days compared to 3.6 (untreated). Only the combination treatment showed transient tumor shrinkage on the day after PRRT and a reduced growth afterwards with a doubling time of 5.8 days (Fig. 5C, Supplementary Fig. 4B).

With respect to median survival, the H69-tumor bearing mice showed a significant survival benefit in the combination therapy groups (median survival not reached with 4/6 (Ruc + PRRT) and 6/6 (Ola + PRRT) animals still alive at day 25; both p = 0.0007 vs. untreated). PRRT monotherapy also showed a significant benefit (24 days; p = 0.0446 vs. untreated) over both PARPi monotherapies (19 and 21 days; n.s. vs. untreated) compared to the untreated control (18 days) (Fig. 5D). In mice bearing H446 tumors, the combination therapy led to an increase in median survival (16 days) compared to untreated animals (12 days), while no survival benefit could be observed for Rucaparib (13 days) or PRRT (11 days) monotherapy (Fig. 5E).

**Fig. 6:**
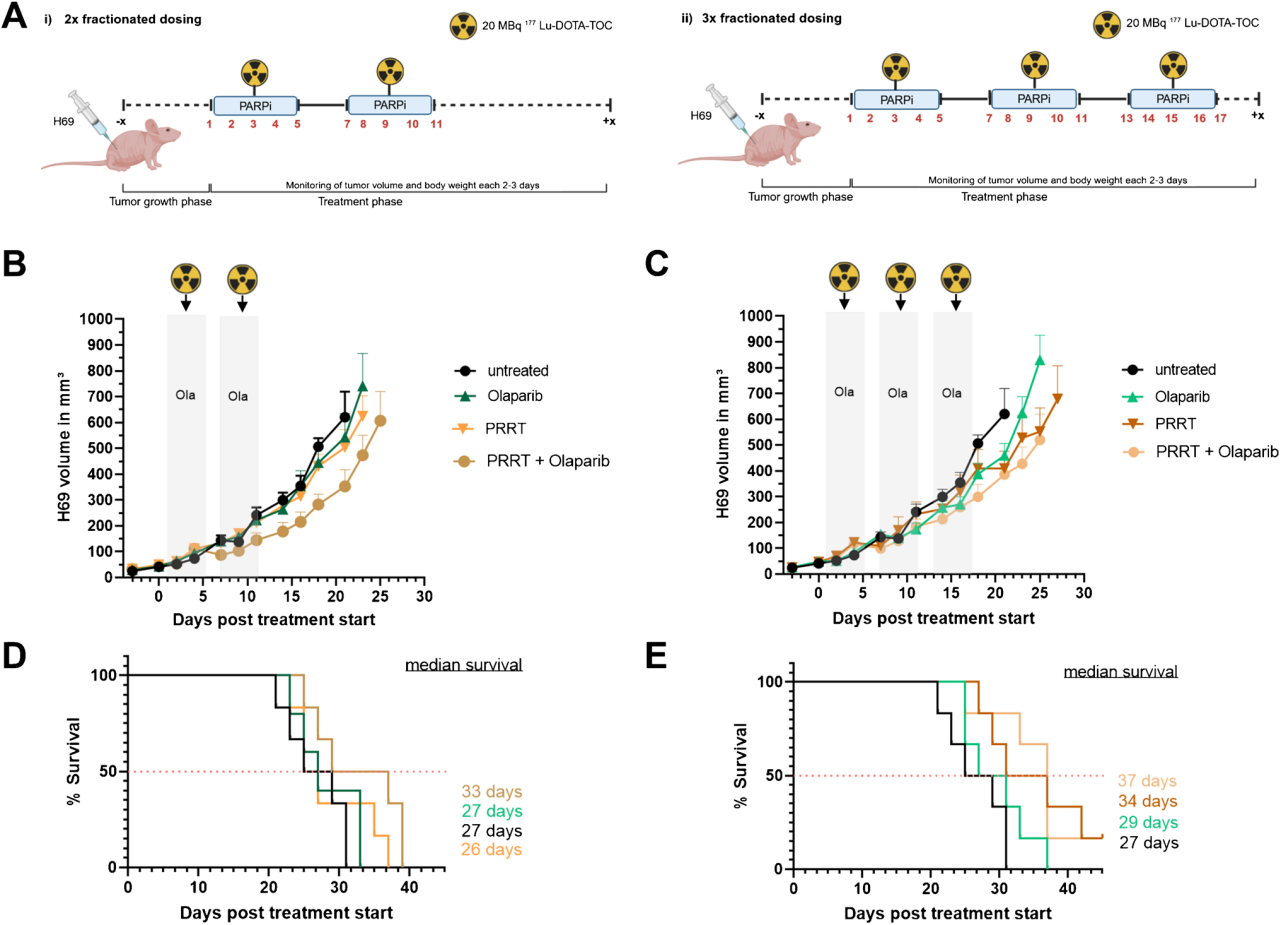
In vivo treatment study for fractionated PRRT application. **(A)** Application schedules of (i) two- and three-fractionated dosing cycles using H69 tumor bearing mice. Volumes of H69 tumors in the **(B)** two-fractionated dosing arm and **(C)** three-fractionated dosing schedule. Grey areas depict days of Olaparib therapy. Radioactivity signs indicate the days of PRRT. Tumor volumes are given as mean ± SEM. The curves end when the first animal per group reached a tumor volume of 1000 mm.^3^ with a maximal size in one dimension of ≤ 15 mm. Kaplan–Meier plots of the two-fractionated **(D)** and three-fractionated **(E)** dosing arms. The observation time ended 4 weeks after the last Olaparib injection (45 days post therapy start)

Subsequently, we tested the effects of fractionation of the PRRT into multiple doses of 20 MBq in combination with Olaparib in the H69 model. The treatment schedules for two- and three-dose fractionation arms are shown in Fig. 6A and 6B. SPECT imaging confirmed that uptake of [^177^Lu]Lu-DOTA-TOC was sustained during week 2 and 3 of treatment (Supplementary Fig. 5). Tumor growth analysis (Fig. 6B and 6C) showed that monotherapies did not lead to tumor control. Combination therapy in both dosing regimens showed a slightly delayed tumor growth compared to the untreated control or either monotherapy, with no differences between the 2 × and 3 × fractionation. Single mouse analysis indicated a heterogeneous response within different treatment groups (Supplementary Fig. 6A). The median survival (Fig. 6D and 6E) was significantly extended in the three-fractionation combination (37 days, p = 0.0071 vs. untreated) group compared to the untreated group (27 days), while the two-fractionation combination group showed a less pronounced extension of 6 days (33 days, p = 0.1109 vs. untreated). In the two-fractionated dosing arm, either monotherapy did not lead to a survival benefit compared to untreated tumor-bearing mice. In contrast, a mild prolongation of animal survival was observed with 3 × PRRT (34 days), but not with 3 × Olaparib monotherapy.

We did not observe any relevant acute toxicities during the single and fractionated dose studies. No weight loss (Supplementary Fig. 6B and 7) or other signs of distress were observed.

### Histopathological analysis after in vivo treatment

**Fig. 7:**
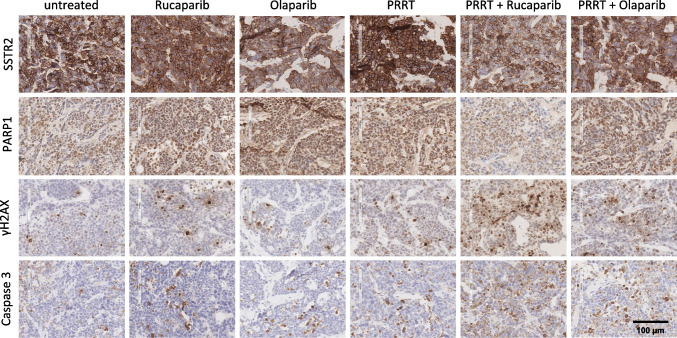
Immunohistochemistry of H69 tumors. Exemplary stainings of H69 tumors obtained from the single dose PRRT study (*n* = 1 animal/group) for SSTR2, PARP1, γH2AX (DNA damage marker) and cleaved caspase 3 (apoptosis marker) are displayed. Scale bar = 100 µm. Brown color indicates positivity for the respective marker

We conducted an exploratory histological analysis in the H69 cohort of the single dose PRRT study with one animal per group on day 3 after PRRT (Fig. 7, Supplementary Fig. 8). Histology showed some heterogeneity of SSTR2 expression within single tumors. PARP1 expression seemed to be increased in all treatment groups compared to the untreated tumor except for the Rucaparib combination group. γH2AX (DNA damage) and cleaved caspase 3 (cell death) had the highest expression in the Rucaparib combination group, followed by Olaparib combination as well as Rucaparib and PRRT monotherapies.

Histopathological analysis by an experienced veterinary pathologist on day 3 after therapy showed no relevant safety signals in bone marrow and kidney (Supplementary Fig. 9). However, these results have to be considered incidental, since we only analyzed one animal per group. Therefore, we cannot determine if we observed actual biological effects of the treatment or naturally occurring variabilities of biomarker expression between different individuals. Future, larger scale studies are needed to elucidate this question.

## Discussion & conclusion

In this study, we showed that PARP inhibition can sensitize SCLC cells and xenografts with low SSTR2 expression to [^177^Lu]Lu-DOTA-TOC PRRT. The addition of either Olaparib or Rucaparib lowered the required PRRT dose to reduce viability to 50% by up to 67-fold in vitro and caused a significant delay in tumor growth and mouse survival in vivo. Mechanistically, the observed sustained DNA damage supports the hypothesis that inhibition of PARP lead to the accumulation of PRRT-induced DNA damage.

Our model system consisted of two SCLC cell lines with distinctly lower SSTR2 expression than AR42J cells [22, 23], reflecting the typical clinical scenario of lower SSTR2 expression in SCLC compared to GEP-NETs. Neither H69 nor H446 cell lines have a reported HR deficiency or hypersensitivity to PARP inhibition. In fact, Lok et al. [24] described H69 and H446 as relatively resistant to PARP inhibition, with low expression levels of markers for PARPi sensitivity (e.g. SLFN11). Hence, the cell lines represent a relevant model to test the effect of PARPi radiosensitization on PRRT efficacy.

The cell line with lower uptake of [^177^Lu]Lu-DOTATOC (H446) showed a higher sensitivity to PRRT and PRRT + PARPi in in vitro viability assays, compared to H69 cells, in line with a previous study reporting intermediate (H446) to low (H69) radiosensitivity [25]. However, these differences were not observed in all in vitro assays or in vivo, where H69 responded better than H446 cells. We attribute this to the complex setting of the combination therapy, where a combination of several factors determines the effectiveness of therapy. Our histological assessment suggests that SSTR2 expression in the H446 model was not only lower per cell, but also more spatially heterogenous in xenografts. Thus, our results indicate that potentiation of radiotherapy is possible, even when radiosensitivity and PARPi sensitivity is low and SSTR2 expression is heterogenous.

Synergy between PARP inhibition and PRRT was also observed by Nonnekens et al. [26], where Olaparib enhanced [^177^Lu]Lu-DOTA-TATE induced cell death in U2OS osteosarcoma cells. The potentiation of the cytotoxicity of [^177^Lu]Lu-DOTA-TATE by PARPi was also reported by Purohit et al. [27], which showed that PARP inhibition increased apoptosis and decreased proliferation in combination with [^177^Lu]Lu-DOTA-TATE in BON1 and NCI-H727 cells. In vitro studies using the AR42J model demonstrated that the combination of [^177^Lu]Lu-DOTA-TATE and the PARPi Talazoparib led to increased numbers of DNA double strand breaks compared to [^177^Lu]Lu-DOTA-TATE alone. These results were confirmed in vivo in the AR42J model, where the combination of [^177^Lu]Lu-DOTA-TATE and Talazoparib significantly improved the anti-tumor efficacy [28]. An increased efficacy of the combination of [^177^Lu]Lu-DOTA-TATE and Olaparib has also been observed by Feijtel et al. using mice bearing CA20948 rat pancreatic tumor-bearing mouse models [29]. Of note, no radiosensitization by Olaparib was observed in the H69 tumor-bearing mouse model [29]. Compared to our study, Feijtel et al. used a lower PRRT dose (30 MBq vs. 40 MBq) and a higher Olaparib dose (50 mg/kg vs. 10 mg/kg), which offers a potential explanation for the different results. Interestingly, a previous study of PRRT in an H69-bearing mouse model with ^177^Lu-DOTA-TATE reports a moderate tumor growth delay after a single dose of 45 MBq, which is consistent with our findings. In this study, they also treated with higher doses of 60 and 120 MBq and found dose-dependent efficacy [30].

Additionally, our fractionated dose study showed that fractionation into smaller doses (20 MBq per cycle) markedly reduced the radiosensitization effect and did not lead to enhanced therapeutic or prolonged effects on tumor growth delay and survival compared to the single PRRT study. This underlines that a high PRRT dose seems necessary to enable radiosensitization effects of PARPi, especially when SSTR2 expression is low and cellular proliferation is high. This is supported by Schmitt et al., where 2 × 45 MBq fractionation of ^177^Lu-DOTA-TATE in H69-tumor bearing mice showed strongly increased tumor regression compared to 1 × 45 MBq and even 1 × 120 MBq [30]. Hence, it seems to be advised to not lower the PRRT dose in the fractionation setting in low SSTR2 expressing cancers in the combination treatment setting.

As combination of antitumor therapies often leads to an increase in adverse events, dose-limiting toxicities have to be observed carefully. Combination therapy of ^177^Lu-PRRT and PARPi has been translated to early-stage clinical trials in metastatic castration resistant prostate cancer [31]. In the ongoing phase 1 trial (LuPARP), interim results suggest that the treatment was generally well tolerated with no observed dose-limiting toxicities [31]. Main side effects were hematological toxicities, as expected, which could be treated well with supportive care until they resolved. In addition, the combination of Olaparib and the chemotherapeutic agent Temozolomide was found to be safe in recurrent SCLC in a phase I/II study, where the majority of side effects were grade 1 and 2 myelosuppression, fatigue, nausea and vomiting [32]. The most common grade 3 and 4 adverse events were thrombocytopenia and neutropenia. These clinical observations are in line with our preclinical study which indicated no severe toxicity of the combination therapy.

In conclusion, the addition of PARPi markedly improved the potency of SSTR2-targeted PRRT in SCLC models in SSTR2 low-expressing tumors. Further evaluation in humans seems justified based on the results as novel treatment options for SCLC are urgently needed.

## Supplementary Information

Below is the link to the electronic supplementary material.

Supplementary file1 (DOCX 21 KB)

Supplementary file2 (PDF 534 KB)

Supplementary file3 (PDF 122 KB)

Supplementary file4 (PDF 146 KB)

Supplementary file5 (PDF 115 KB)

Supplementary file6 (PDF 140 KB)

Supplementary file7 (PDF 654 KB)

Supplementary file8 (PDF 114 KB)

Supplementary file9 (PDF 773 KB)

Supplementary file10 (PDF 428 KB)

## Data Availability

The datasets generated during and/or analysed during the current study are available from the corresponding author on reasonable request.
