# Supplementary material for: Combining [177Lu]Lu-DOTA-TOC PRRT with PARP inhibitors to enhance treatment efficacy in small cell lung cancer
Source: Eur J Nucl Med Mol Imaging. 2024 Jul 18;51(13):4099–110. doi: 10.1007/s00259-024-06844-1 (PMC11527929; doi:10.1007/s00259-024-06844-1)
Supplement: Supplementary file 1 — Supplementary file1 (DOCX 21 KB) [file 259_2024_6844_MOESM1_ESM.docx]

**Supplementary Figure legends:**

**Supplementary Figure 1**. Chemical structures of the unmodified **(A)** and Sulfo-Cy5 labelled sst-antagonist **(B)**. **(C)** Quality control of (B). P=product, M=molecular weight of the product; HPLC-chromatogram w/ 10-90% MeCN + 0.1% TFA in 15 min, at 220 nm, 254 nm, and 647 nm and LC-MS chromatogram of the same run. Product peak at *t*_R_  = 8.6 min. MS, ESI+: m/z calculated 2784.6: found m/z 1859 (2(M+3H^+^), 1394 (M+2H^+^), 930 (M+3H^+^).

**Supplementary Figure 2.** Tumor volumes on the day before start of treatment. Mice were randomly assigned to different treatment cohorts (n=5-6 per group) demonstrating uniform initial mean tumor volumes of each treatment group in the respective model. **(A)** Tumor volumes of H69 and **(B)** H446 xenografts of the single dose application regime. **(C)** Tumor volumes of H69 xenografts of the fractionated dosing schedule.

**Supplementary Figure 3**. SPECT imaging and biodistribution. **(A)** Maximum intensity projections of mice bearing subcutaneous H69 (left) and H446 (right) xenografts (white dashed circle). Data was acquired 1 h, 24 h and 72 h after a single dose of ca. 25 MBq [^177^Lu]Lu-DOTA-TOC. Mice are shown in ap view with xenografts on the right shoulder. Exemplary animal from n=3/group shown. **(B)** Quantification of tumor uptake from 72 h p.i. SPECT images. **(C)** Biodistribution in H69 and H446 xenograft bearing mice 72 h after a single dose of 40 MBq [^177^Lu]Lu-DOTA-TOC. Values are mean ± standard deviation from n=3 animals per group.

**Supplementary Figure 4**. Individual tumor volumes of single PRRT mice. **A** Individual tumor volumes of H69 xenograft bearing mice without treatment, with treatment with PARPi (rucaparib, olaparib), PRRT or the combination of PARPi and PRRT. **B** Individual tumor volumes of H446 xenograft bearing mice without treatment, treatment with rucaparib, PRRT or the combination of rucaparib and PRRT.

**Supplementary Figure 5.** SPECT imaging of H69 bearing mice in the fractionated dosing therapy study. Imaging was performed for 60 min within the 2^nd^ and 3^rd^ application cycle 24 h after i.v. injection of approx. 20 MBq [^177^Lu]Lu-DOTA-TOC (n=2). Tracer accumulation in SSTR2-expressing H69 tumors and kidneys is shown as indicated by arrows.

**Supplementary Figure 6.** Individual tumor volumes of the fractionated dose study. **(A)** Overview of individual tumor volumes of H69 xenografts and **(B)** individual body weights of H69 bearing mice of the different cohorts of the fractionated dosing therapy study starting on the day before treatment start (day 0).

**Supplementary Figure 7.** Overview of individual body weights of **(A)** H69 and **(B)** H446 xenografts of the different cohorts within the single dose therapy study starting the day of tumor inoculation**.**

**Supplementary Figure 8**. Exemplary overview sections of H69 xenografts explanted after 5 days of PARPi treatment/3 days after PRRT treatment stained for SSTR2, PARP1, yH2AX (DNA damage) and cleaved caspase3 (apoptosis).

**Supplementary Figure 9**. Exemplary section of H&E stained kidney and bone marrow (femur) samples of mice treated with olaparib, PRRT and the combination compared to untreated control at 20x magnification. Animals treated with Rucaparib alone showed slight bone marrow hypercellularity, while the combination of Rucaparib and PRRT showed slight hypocellularity. Animals in the Rucaparib monotherapy as well as the Olaparib combination groups showed low levels of angiectasis in the bone marrow. In the kidney tubule, dilatation was observed in very small amounts in the Olaparib combination group.
