# Supplementary figures and images for: Combining [177Lu]Lu-DOTA-TOC PRRT with PARP inhibitors to enhance treatment efficacy in small cell lung cancer
Source: Eur J Nucl Med Mol Imaging. 2024 Jul 18;51(13):4099–110. doi: 10.1007/s00259-024-06844-1 (PMC11527929; doi:10.1007/s00259-024-06844-1)

Supplementary Figure 1

A

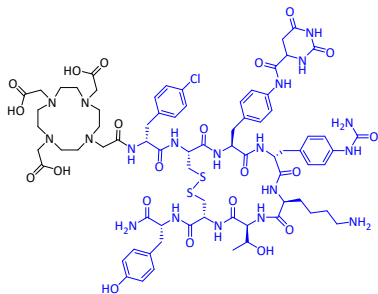

B

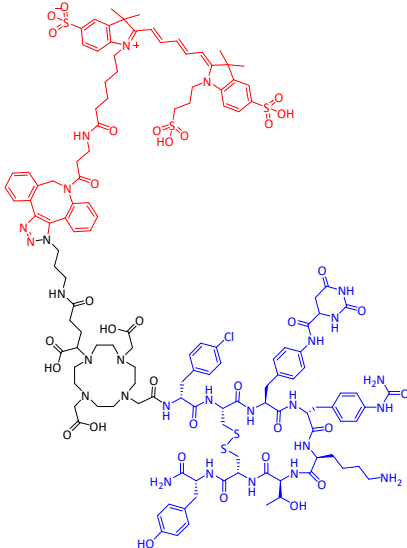

C

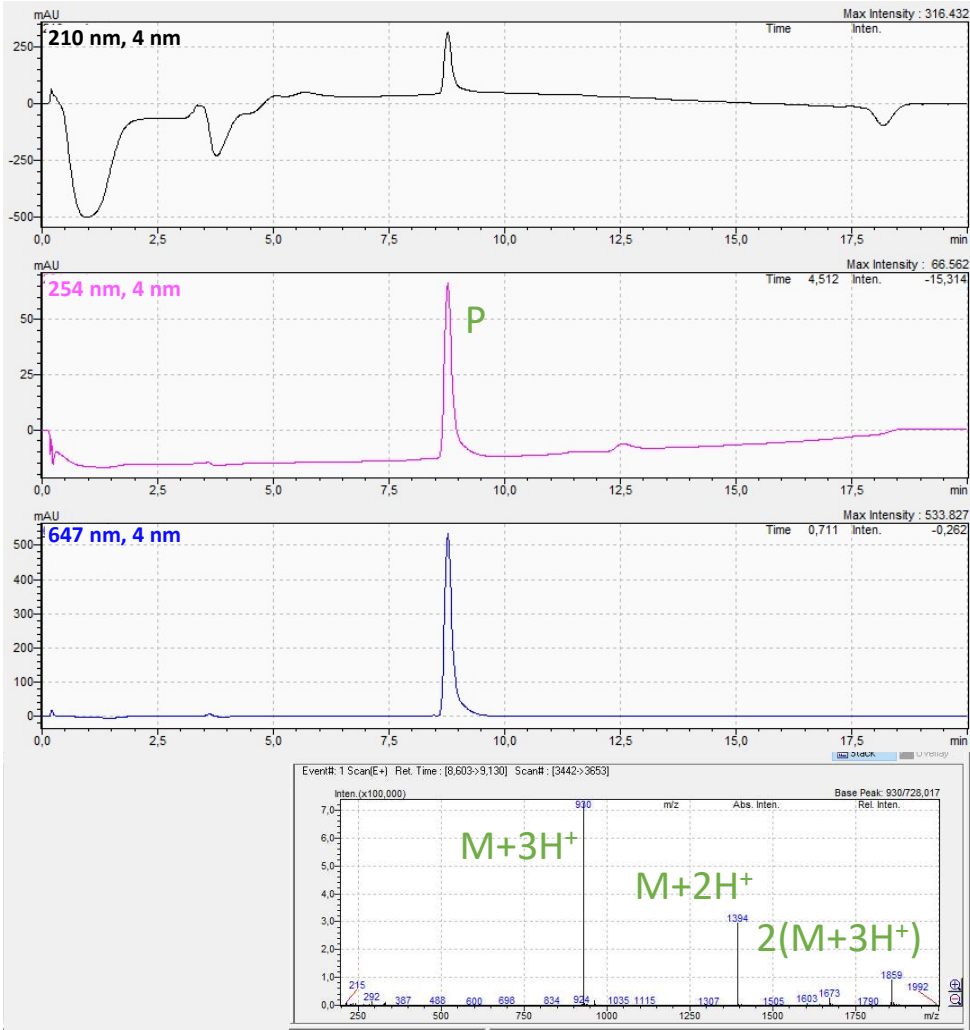

Supplement: Supplementary file 2 — Supplementary file2 (PDF 534 KB) [file 259_2024_6844_MOESM2_ESM.pdf]

Supplementary Figure 3

A

H69

23.0  
MBq

1 h

24 h

72 h

H446

25.0  
MBq

1 h

24 h

72 h

10

%ID/g

0

B

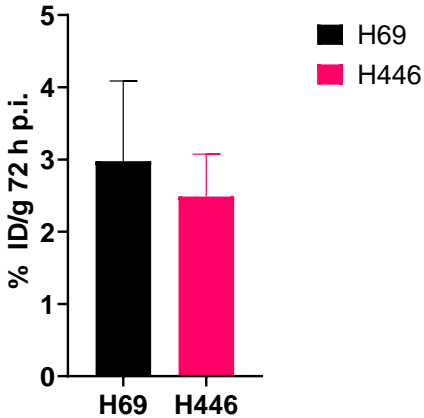

C

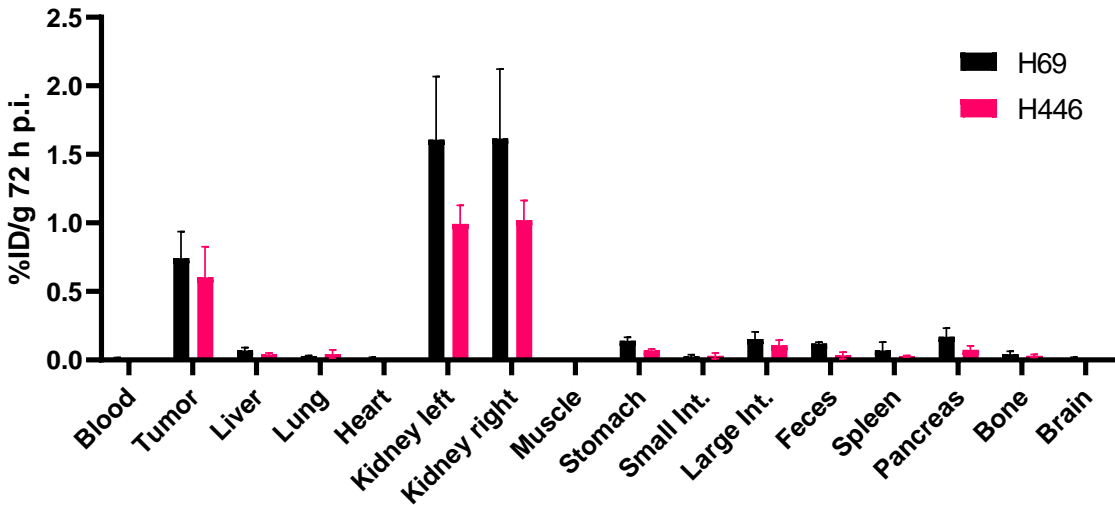

Supplement: Supplementary file 4 — Supplementary file4 (PDF 146 KB) [file 259_2024_6844_MOESM4_ESM.pdf]

Supplementary Figure 5

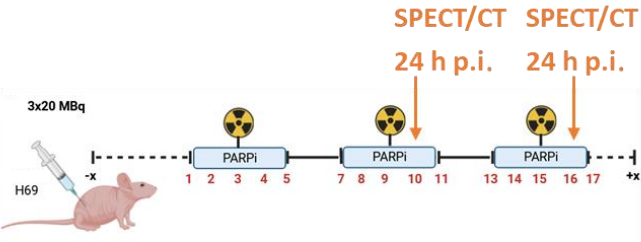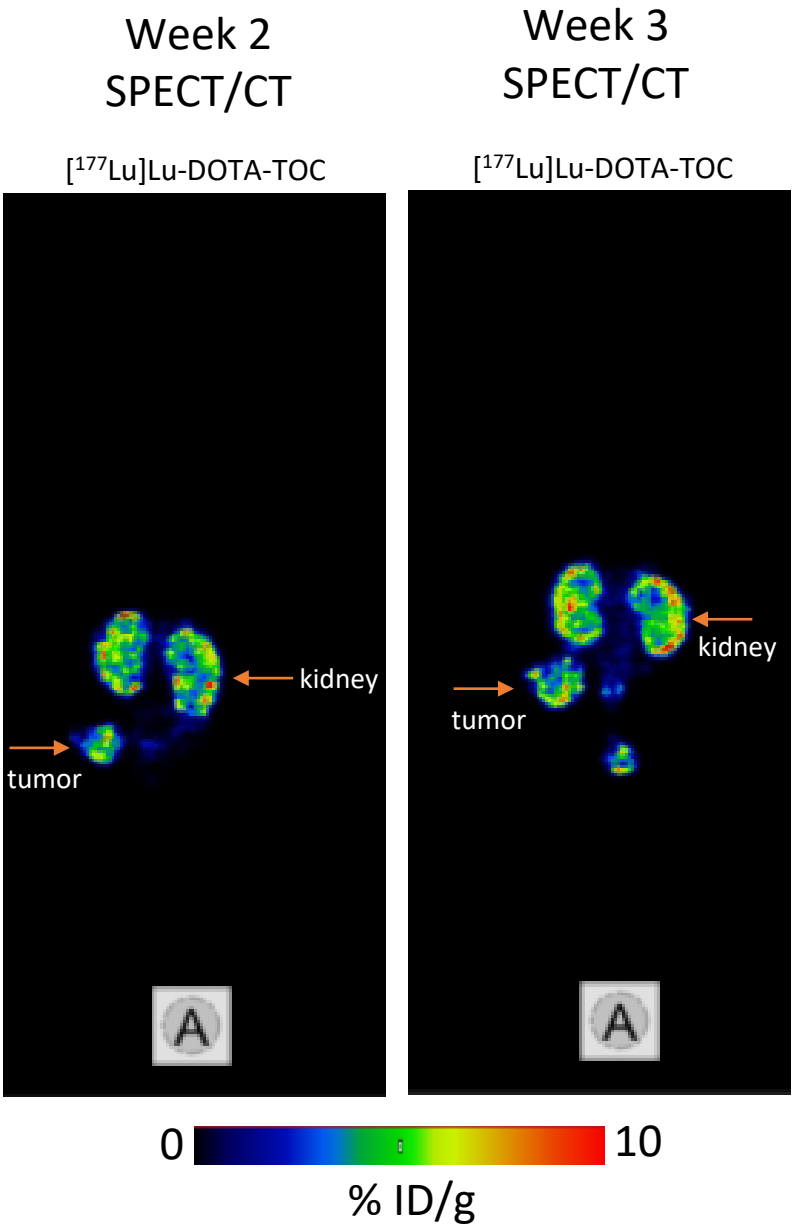

Supplement: Supplementary file 6 — Supplementary file6 (PDF 140 KB) [file 259_2024_6844_MOESM6_ESM.pdf]

Supplementary Figure 6

A

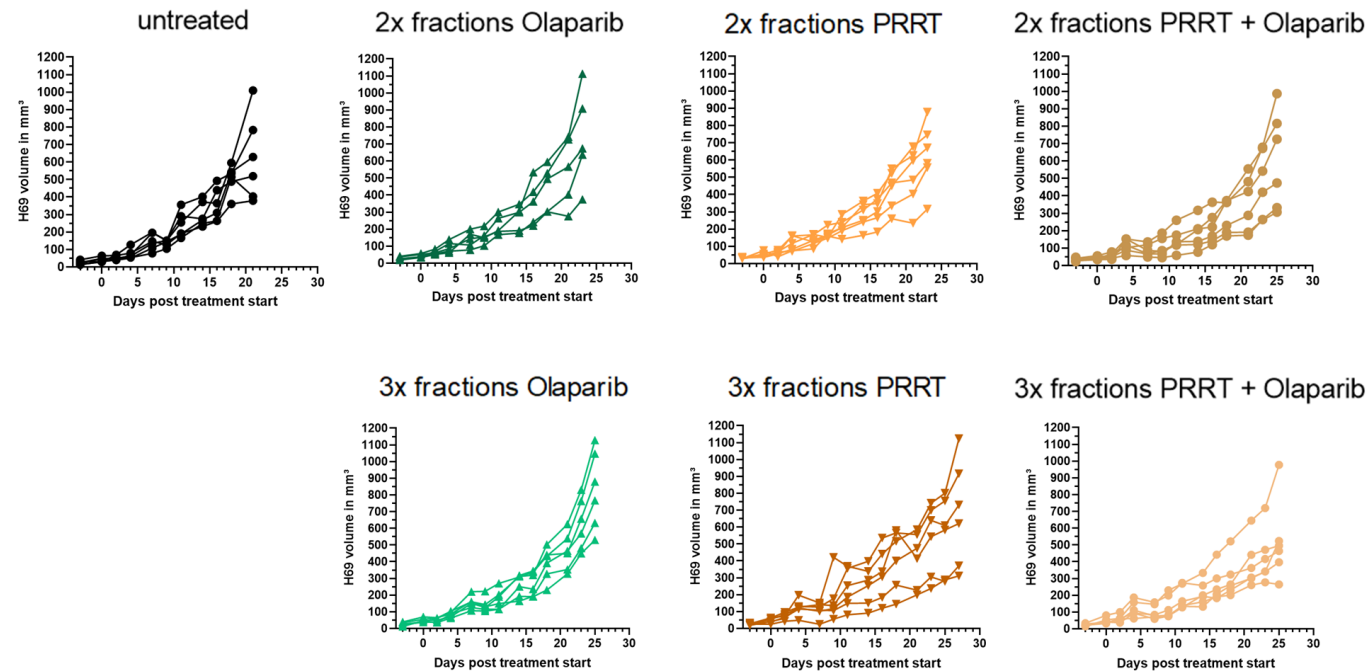

B

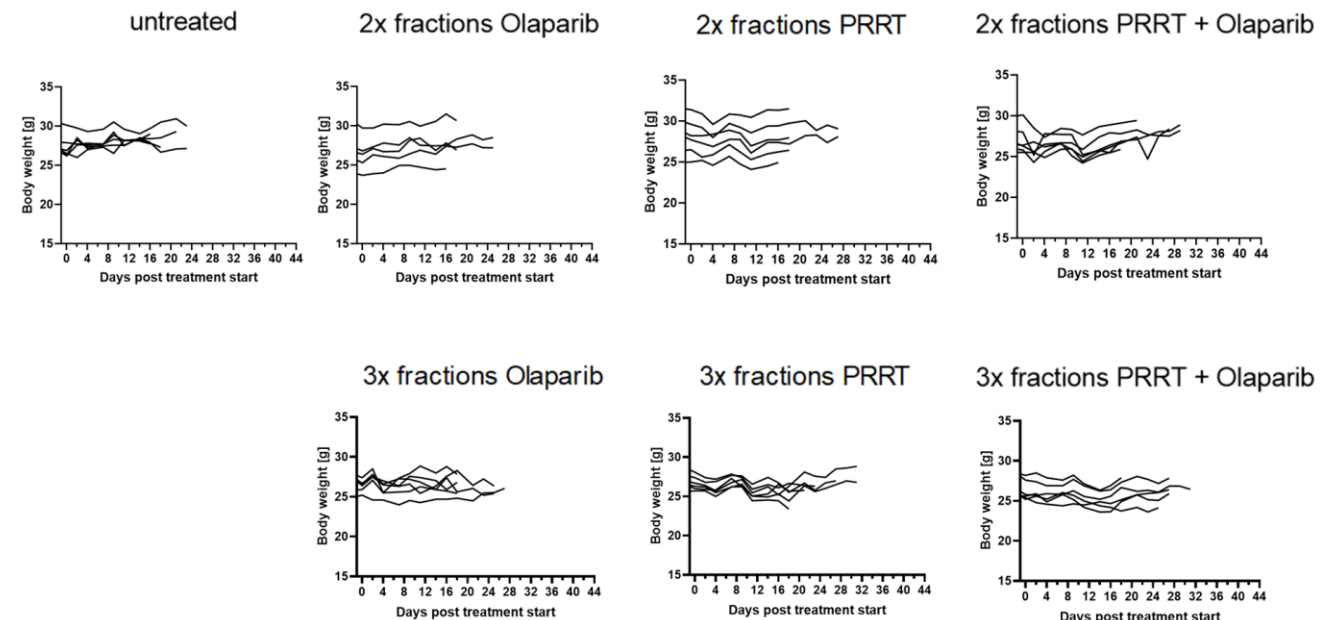

Supplement: Supplementary file 7 — Supplementary file7 (PDF 654 KB) [file 259_2024_6844_MOESM7_ESM.pdf]

Supplementary Figure 8

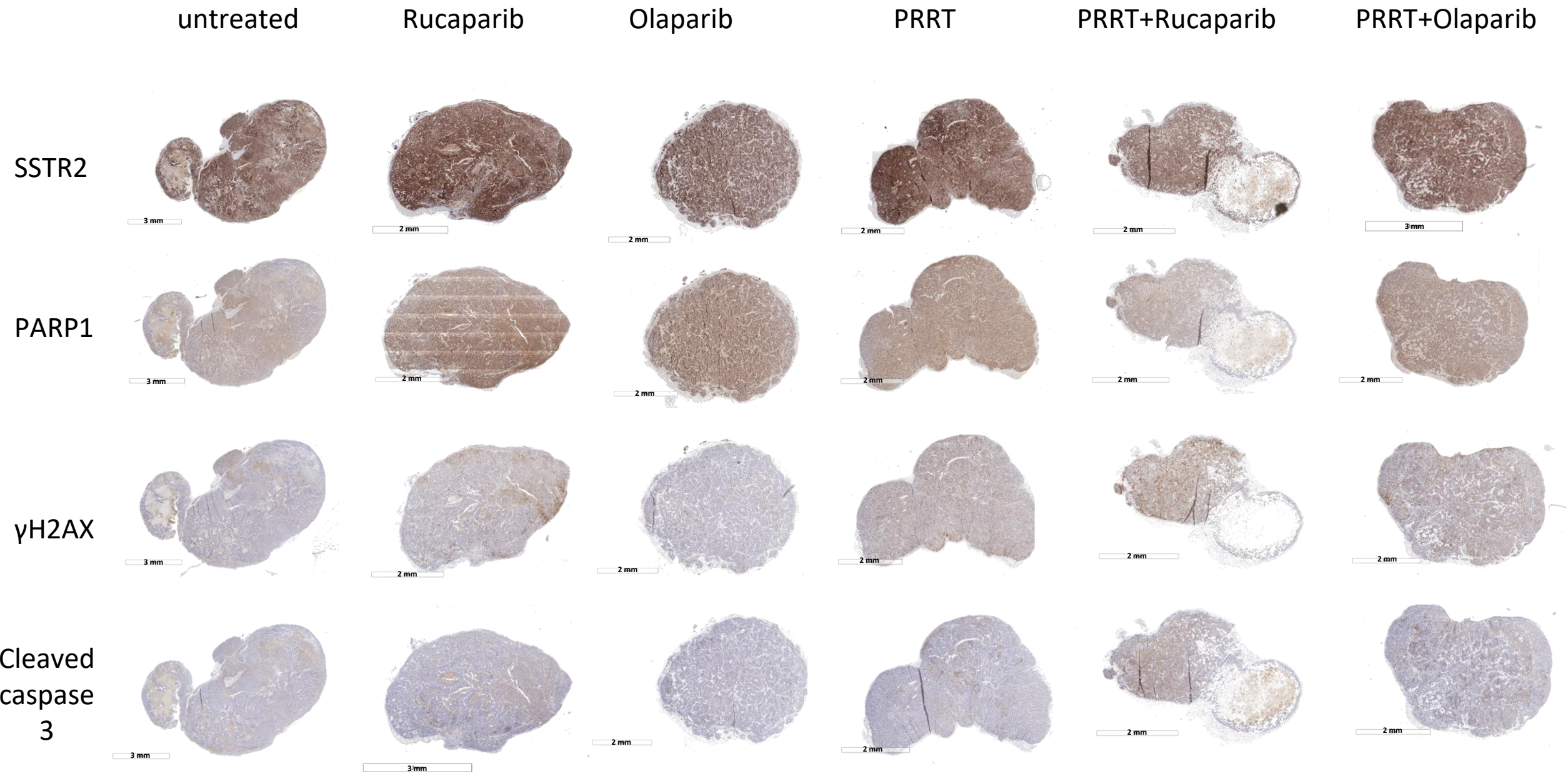

Supplement: Supplementary file 9 — Supplementary file9 (PDF 773 KB) [file 259_2024_6844_MOESM9_ESM.pdf]

Supplementary Figure 9

**B**

Bone marrow

Untreated

Olaparib

PRRT

PRRT + Olaparib

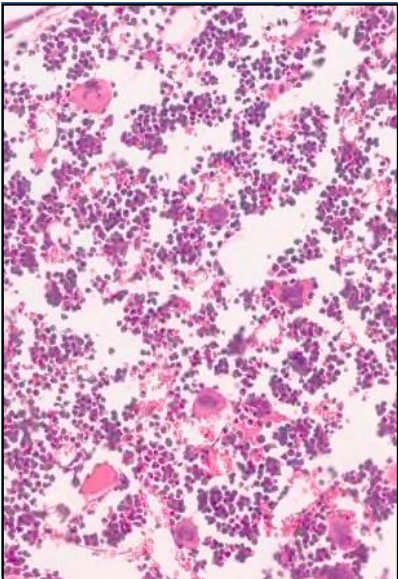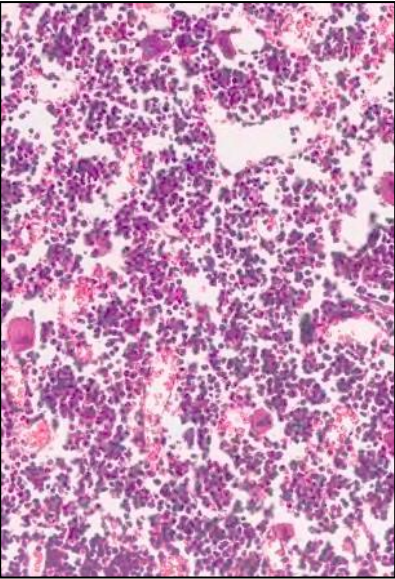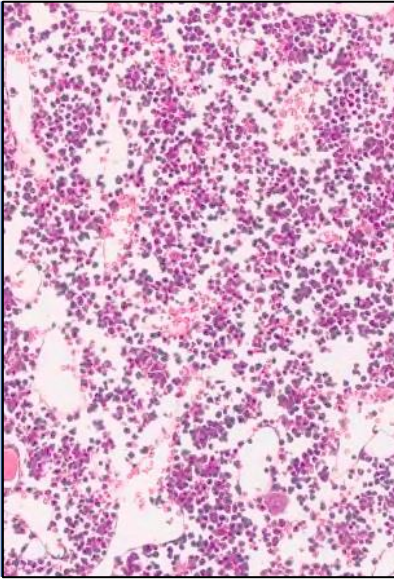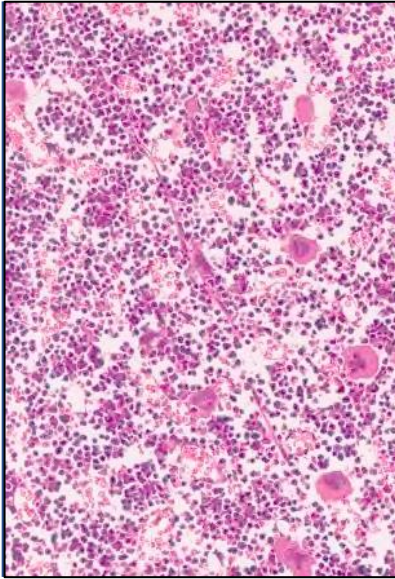

Kidney

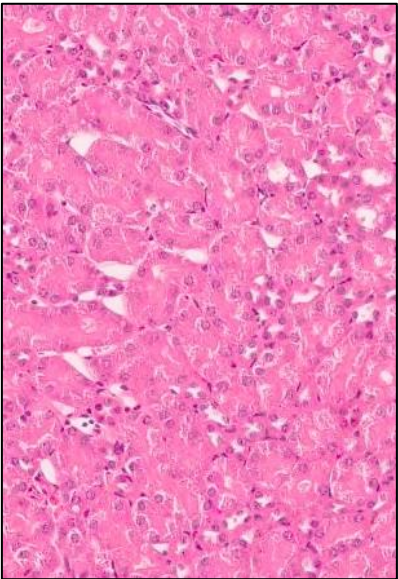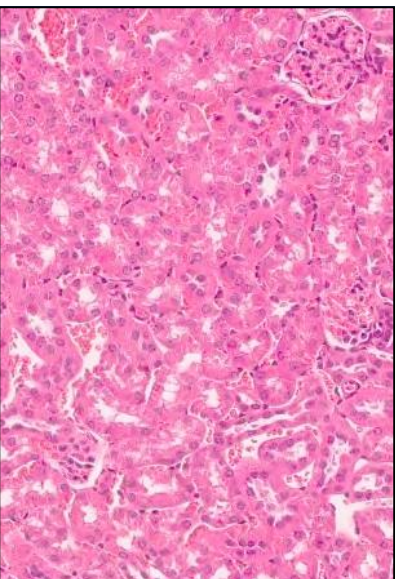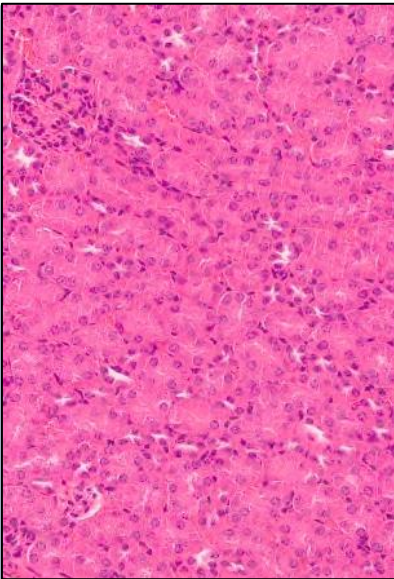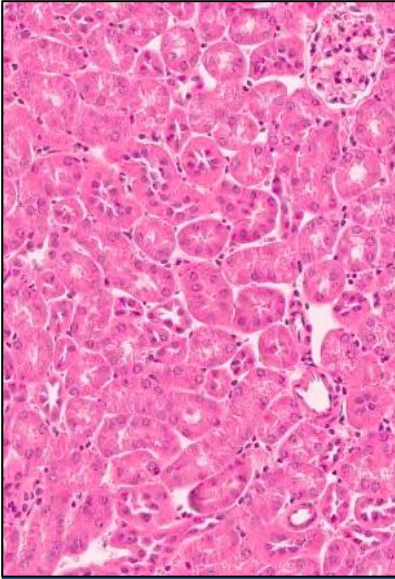

Supplement: Supplementary file 10 — Supplementary file10 (PDF 428 KB) [file 259_2024_6844_MOESM10_ESM.pdf]
