# Supplementary material for: Combining [177Lu]Lu-DOTA-TOC PRRT with PARP inhibitors to enhance treatment efficacy in small cell lung cancer
Source: Eur J Nucl Med Mol Imaging. 2024 Jul 18;51(13):4099–110. doi: 10.1007/s00259-024-06844-1 (PMC11527929; doi:10.1007/s00259-024-06844-1)

Supplementary Figure 7

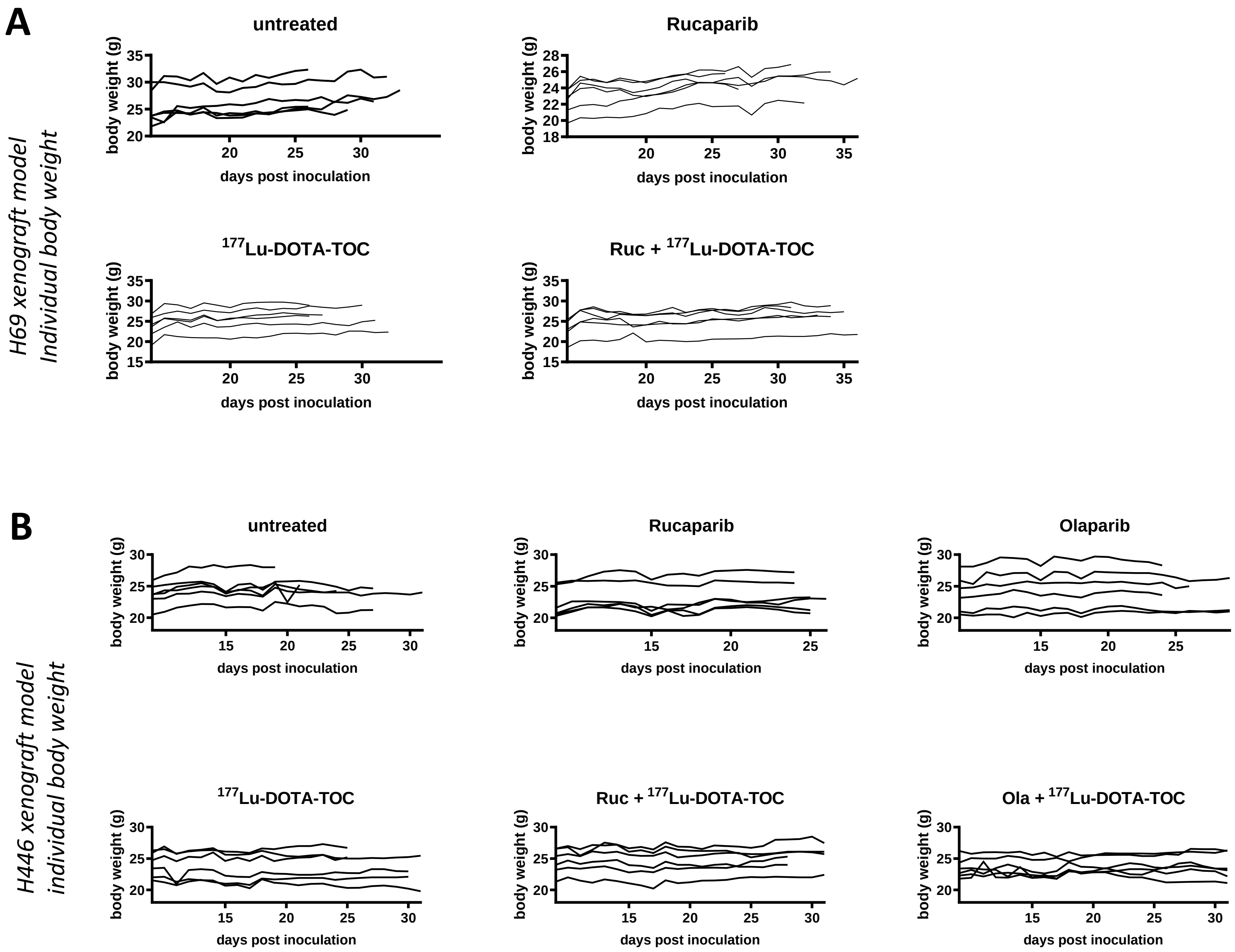

Supplement: Supplementary file 8 — Supplementary file8 (PDF 114 KB) [file 259_2024_6844_MOESM8_ESM.pdf]
